# Genetic association of RIT2 rs12456492 polymorphism and Parkinson’s disease susceptibility in Asian populations: a meta-analysis

**DOI:** 10.1038/srep13805

**Published:** 2015-09-03

**Authors:** Yanjun Lu, Wei Liu, Kun Tan, Jing Peng, Yaowu Zhu, Xiong Wang

**Affiliations:** 1 Department of Laboratory Medicine, Tongji Hospital, Tongji Medical College, Huazhong University of Science and Technology, Wuhan 430030, China; 2 Department of Public Health, Tongji Hospital, Tongji Medical College, Huazhong University of Science and Technology, Wuhan 430030, China; 3 Department of Infection Control, Tongji Hospital, Tongji Medical College, Huazhong University of Science and Technology, Wuhan 430030, China

## Abstract

Recent studies investigating the association of the Ras-like without CAAX 2 (*RIT2*) polymorphism, rs12456492, with Parkinson’s disease (PD) are controversial. We performed a meta-analysis to study the association between rs12456492 and PD susceptibility in Asian populations. Literature searches of PubMed and Embase were performed up to June 3, 2015, and the strength of the association between rs12456492 and PD was evaluated by odds ratios (OR) and 95% confidence intervals (CI). Four studies conducted between 2013 and 2015, comprising 2017 PD cases and 2010 controls, were included in the meta-analysis. Significant association of rs12456492 with PD was found in the dominant (GG + AG vs. AA: OR = 1.26, 95% CI = 1.20–1.44, P = 0.00) and additive models (GG vs. AA: OR = 1.38, 95% CI = 1.03–1.83, P = 0.030). Although sensitivity analysis found that the overall result was stable only in the dominant genetic model, a publication bias was also detected. Therefore, the results should be treated with caution. The current meta-analysis suggested that rs12456492 might be associated with increased PD risk in Asian populations, but studies using larger sample sizes and different ethnic populations will be needed to further confirm this association.

Parkinson’s disease (PD) is an age-related, progressive, complex neurodegenerative disease, which significantly affects quality of life and affects approximately 1% of people over 60 years of age. The disease is characterized behaviorally by motor and non-motor symptoms and pathologically by aggregations of the α-synuclein protein^1^,^2^. PD involves both genetic and environmental factors, and associations between hereditary PD and mutations in certain genes have been identified, including *LRRK2*, *PARKIN*, *DJ1*, *ATP13A2*, *VPS35*, *PINK1,* and *SNCA*^3^,^4^,^5^. However, these mutations account for only a small percentage of PD cases, and the majority of PD cases are the sporadic form of the disease, indicating that additional genes may also contribute to the risk of PD.

Recently, several genome-wide association studies (GWAS) have identified numerous genes associated with sporadic PD, including *SNCA*, *GBA*, *MAPT*, and *HLA*^6^,^7^. In 2012, Pankratz *et al.* identified an association between a polymorphism in Ras-like without CAAX 2 (*RIT2*), rs12456492, and PD risk in a large White population when performing a meta-analysis of GWAS results^8^. Several studies have shown results that were similar to those of Pankratz *et al.*^9^,^10^,^11^; however, Lin *et al.* found no association between rs12456492 and PD in a Taiwanese population^12^. To derive a more precise estimation of the association between rs12456492 and PD in Asian populations, we carried out the current meta-analysis, aiming to study the role of rs12456492 in PD pathogenesis and to explain the possible reasons for the controversial results.

## Methods

### Search strategy

Literature searches were performed using PubMed and Embase. The search strategy was based on the following search terms, “Parkinson’s disease or PD” and “Ras-like without CAAX 2 or RIT2 or Rin or rs12456492” and “polymorphism or mutation or variation or SNP”, with the last report up to June 3, 2015. All eligible studies were retrieved, and their bibliographies were examined for relevant publications.

### Inclusion criteria

The inclusion criteria for studies were as follows: (1) association shown between rs12456492 and PD risk in Asian populations; (2) case-control design; and (3) available phenotype or allele frequencies of rs12456492 in cases and controls. Abstracts from conferences, studies without sufficient data for odds ratios (OR) and 95% confidence intervals (CI), republished or duplicate studies, and reviews were excluded.

### Data extraction

Data from eligible studies were extracted by Yanjun Lu and Wei Liu, according to the above inclusion and exclusion criteria. The following information was collected from each study: the first author, year of publication, country, ethnicity of the sample population, sample size, phenotype distribution, and minor allele frequency (MAF).

### Statistical analysis

Data were analyzed by STATA software, version 11.0 (STATA Corp., College Station, TX, USA). Hardy Weinberg equilibrium (HWE) in the control group of each study was examined, and P < 0.05 was considered as departure from HWE. The association between rs12456492 and risk of PD was evaluated by pooled OR and 95% CI. Four genetic models, including the allelic (G vs. A), dominant (GG + AG vs. AA), recessive (GG vs. AG + AA), and additive (GG vs. AA), were used to analyze the association. The significance of the pooled OR was assessed by the Z test, and P < 0.05 was considered as statistically significant. The Q-test and I^2^ tests were used to investigate the heterogeneity between studies. If P > 0.10 for the Q-test, or if I^2^ < 50%, the fixed-effect model was used to calculate the pooled ORs; if P < 0.10 for the Q-test, or if I^2^ > 50%, the random-effect model was applied. Sensitivity analysis was carried out by sequentially omitting one study at a time to estimate the stability of the result. Publication bias among studies was determined using Begg’s test and Egger’s test.

## Results

### Study selection and characteristics

The selection process of studies included in this meta-analysis is shown in Figure S1. The initial search found 42 studies (PubMed: 33, Embase: 9). Of these, four studies in Embase were also found in PubMed; therefore, 38 total studies were identified. After a careful abstract or full-text review, one review, 30 irrelevant studies, and two articles without sufficient genotype information^8^,^13^ were excluded. The remaining five studies were selected, and the data were extracted from these. However, a study conducted by Nie *et al.* departed from HWE (P = 0.03), and their data were therefore excluded from further meta-analysis^11^. Finally, four studies published between 2013 and 2015 comprising 2017 PD cases and 2010 controls were included in the current meta-analysis^9^,^10^,^12^,^14^. Emamalizadeh *et al.* chose an Asian population in Iran^10^, therefore, all the included four studies were Asian populations. The characteristics and genotypes included in these studies are listed in Tables 1 and 2.

### Meta-analysis results

**Figure 1:**
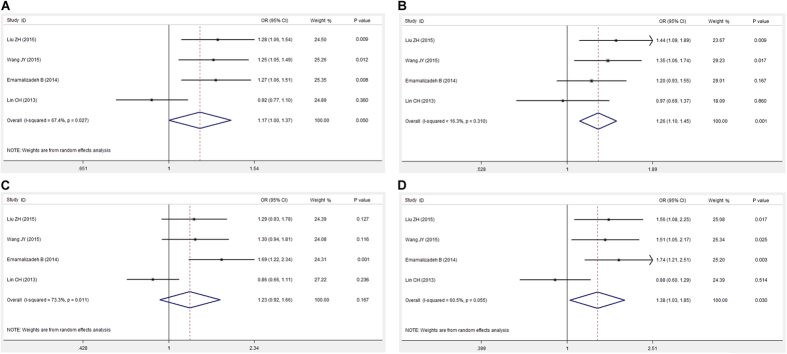
Forest plots for meta-analysis of rs12456492 polymorphism and risk of PD in Asian populations. (**A**) Allelic model (G vs. A). (**B**) Dominant genetic model (GG + AG vs. AA). (**C**) Recessive genetic model (GG vs. AG + AA). (**D**) Addictive genetic model (GG vs. AA).

First, the heterogeneity of rs12456492 in the pooled population was evaluated. The results showed clear heterogeneity in the allelic, recessive, and additive genetic models (Table 3). The ORs and 95% CIs in random- and fixed-effect models were calculated according to the values of P_Q_ and I^2^. We found that rs12456492 was significantly associated with an increased risk of PD in the dominant and additive genetic models in the pooled Asian populations (P < 0.05; Fig. 1).

We performed a stratified analysis by MAF (>0.5 vs. <0.5), and the results showed that rs12456492 was significantly associated with PD in all four genetic models in the subgroup with a MAF < 0.5 (Table 3).

### Heterogeneity test and sensitivity analysis

Meta-regression was performed to explore the source of heterogeneity, considering the publication year and MAF (>0.5 vs. <0.5) as possible covariates. No covariate significantly contributing to the heterogeneity in any genetic model was found (Table 4). However, MAF tended to influence the heterogeneity in the allelic and recessive genetic models (P = 0.094, P = 0.091 respectively). In the overall analysis, rs12456492 was not associated with PD risk in the allelic or recessive genetic models, while in the MAF < 0.05 subgroup, rs12456492 was significantly associated with PD risk in both genetic models. These data suggest that MAF may slightly influence the heterogeneity.

The sensitivity analysis showed that in the allelic and recessive genetic models, after omitting Lin *et al.*’s study, rs1245692 was associated with PD (allelic: OR = 1.27, 95% CI = 1.14–1.40, P = 0.000; recessive: OR = 1.42, 95% CI = 1.17–1.71, P = 0.000). In the additive genetic model, the sensitivity analysis showed that the result was not stable (Liu: OR = 1.33, 95% CI = 0.89–1.98, P = 0.166; Wang: OR = 1.34, 95% CI = 0.89–2.02, P = 0.159; Emamalizadeh: OR = 1.28, 95% CI = 0.90–1.83, P = 0.176). In the dominant genetic model, the sensitivity analysis showed that the result was stable (Liu: OR = 1.21, 95% CI = 1.02–1.41, P = 0.022; Wang: OR = 1.22, 95% CI = 1.04–1.44, P = 0.017; Emamalizadeh: OR = 1.29, 95% CI = 1.10–1.51, P = 0.002; Lin: OR = 1.33, 95% CI = 1.14–1.54, P = 0.000). These data implied that our meta-analysis results were not robust in the allelic, recessive, and additive genetic models but remain stable in the dominant genetic model in Asian populations.

### Publication bias

Publication bias was assessed by Begg’s test and Egger’s test. A publication bias was found in the dominant genetic model. A trending bias also existed in the recessive and additive models (P = 0.087 and P = 0.070 respectively; Table 5). These data indicate that the number of studies analyzed in the current meta-analysis may be insufficient.

## Discussion

RIT2 (formerly named Rin) is a member of the Ras superfamily of small guanosine triphosphate binding proteins localized on the plasma membrane. RIT2 is specifically expressed in neurons, and preferentially expressed in the dopaminergic neurons in the substantia nigra (SN)^15^. RIT2 binds to calmodulin 1, a phosphorylase kinase, which binds to the products of *SNCA* and *MAPT*, susceptibility genes for PD^9^. Furthermore, RIT2 directly interacts with the dopamine transporter and mediates its internalization and functional downregulation via the PKC-regulated pathway^16^. In addition, postmortem examination of PD patients found reduced expression of *RIT2* in the SN compared with unaffected controls^17^. A meta-analysis conducted by Pankratz *et al.* identified rs12456492 as a novel locus for PD^8^. In 2014, Nalls *et al.* performed a large-scale meta-analysis of GWAS data including 13,708 cases and 95,282 controls in a White population. They also identified rs12456492 as a risk locus for PD^13^. Taken together, these data suggest that RIT2 may play an essential role in the pathogenesis and development of PD. Several replication studies have investigated the association of rs12456492 and PD risk in different populations; however, the results were controversial. Thus, we have conducted this meta-analysis to explore the pooled effect size of the association between rs12456492 and PD susceptibility in Asian populations.

We investigated the four selected studies carefully. Significant association of rs12456492 with PD risk was found in the pooled Asian population in the dominant and additive genetic models. Stratified analysis by MAF showed a significant association of rs12456492 with PD risk in all four genetic models in the subgroup with MAF < 0.5. We performed a meta-regression analysis to investigate the source of heterogeneity, although we failed when considering the publication year and MAF. MAF tended to influence the heterogeneity in the allelic and recessive genetic models (P = 0.094, P = 0.091 respectively), as rs12456492 was significantly associated with PD risk in the two genetic models in MAF < 0.5 subgroup. The sensitivity analysis showed that the association between rs12456492 and PD susceptibility was robust only in the dominant genetic model. However, a publication bias existed in the dominant genetic model, and a trending bias existed in the recessive and additive models. Of the four studies included in the meta-analysis, only Lin *et al.* did not find a significant association between rs12456492 and PD risk. As a negative result might not be as readily accepted for publication as a positive result, this could be a contributing factor to the publication bias that we found in the rs12456492 investigation. These results indicated that the number of studies included in the meta-analysis was insufficient. Our meta-analysis suggested that although a significant association of rs12456492 with PD risk was found in the pooled population in some of the genetic models, the results should nonetheless be applied cautiously due to the instability caused by the limited number of studies and the sample size.

Differential associations of rs12456492 with PD susceptibility between different MAF groups might be due to the varying genetic backgrounds and sample sizes. Data from the PubMed SNP database (http://www.ncbi.nlm.nih.gov/projects/SNP/snp_ref.cgi?rs=12456492) showed that the frequency of allele G was 0.375 and 0.339 in Chinese and Japanese populations, respectively. In an Asian population, the highest MAF was 0.5. In the four studies included in the current meta-analysis, three of them had a MAF < 0.5, while Lin *et al.*’s data had a MAF > 0.5. The discrepancy of these results might be due to the genetic heterogeneity between different regions, as Lin *et al.*’s study included a Taiwanese population, while two other studies included a Chinese population from mainland China. This suggests that the complex etiology of PD is influenced by both hereditary and environmental factors. Moreover, in the study by Lin *et al.*, both late-onset PD (62%) and early-onset PD (38%) patients were recruited, and the early-onset PD patients had an age of onset of less than 50 years. However, the mean ages of onset in the other three studies was greater than that in the study by Lin *et al.*, and age might have influenced the heterogeneity and association. Due to insufficient data, we did not examine the contribution of age to heterogeneity in our meta-regression analysis. Therefore, experimental research and human studies with larger sample sizes, different ethnicity, and rigorous design are needed to further clarify this association.

Some limitations were present in this meta-analysis. First, only studies published in English were included. Second, the stratified analysis was performed only by MAF, without considering other factors. Third, the sample size was small. Fourth, only Asian populations were analyzed in the current meta-analysis, as the associations had been found in White populations^8^,^13^. All of these limitations may lead to bias in our results.

In summary, our meta-analysis suggested that rs12456492 was significantly associated with PD in Asian populations. However, additional studies with larger sample sizes, gene-gene and gene-environment interactions in different countries and ethnic populations may be helpful in providing a more reliable estimation of the association between rs12456492 and PD susceptibility.

## Additional Information

**How to cite this article**: Lu, Y. *et al.* Genetic association of RIT2 rs12456492 polymorphism and Parkinson's disease susceptibility in Asian populations: a meta-analysis. *Sci. Rep.* **5**, 13805; doi: 10.1038/srep13805 (2015).

## Supplementary Material

Supplementary Figure S1

## Figures and Tables

**Table 1:** Characteristics of four studies included in this meta-analysis.

Author	Year	Region	Ethnicity	Sample size	
				Case	Control
Liu ZH	2015	China	Asian	460	473
Wang JY	2015	China	Asian	537	517
Emamalizadeh B	2014	Iran	Asian	520	520
Lin CH	2013	Taiwan	Asian	500	500

**Table 2:** Genotype frequencies of rs12456492 in four studies included in this meta-analysis.

Author	Case			Control			MAF		HWE
	GG	AG	AA	GG	AG	AA	Case	Control	
Liu ZH	100	225	135	84	212	177	0.462	0.402	0.141
Wang JY	96	257	184	74	229	214	0.418	0.365	0.317
Emamalizadeh B	111	247	162	72	265	183	0.451	0.393	0.122
Lin CH	171	252	77	189	236	75	0.594	0.614	0.925

**Table 3:** Meta-analysis of rs12456492 polymorphism and risk of PD in Asian populations.

Genetic comparison	P_Q_	I^2^	Random model 95% CI	P_Z_	Fixed model 95% CI	P_Z_
Overall (n = 4)						
G vs. A	0.027	67.4%	1.17 (1.00–1.37)	0.050		
GG + AG vs. AA	0.310	16.3%			1.26 (1.10–1.44)	0.001
GG vs. AG + AA	0.011	73.3%	1.23 (0.92–1.66)	0.167		
GG vs. AA	0.055	60.5%	1.38 (1.03–1.83)	0.030		
MAF < 0.5 (n = 3)						
G vs. A	0.986	0.0%			1.27 (1.14–1.40)	0.000
GG + AG vs. AA	0.623	0.0%			1.33 (1.14–1.54)	0.000
GG vs. AG + AA	0.426	0.0%			1.42 (1.17–1.71)	0.000
GG vs. AA	0.849	0.0%			1.60 (1.30–1.98)	0.000

**Table 4:** Meta-regression of rs12456492 polymorphism and risk of PD in Asian populations.

Genetic comparison	t	P > |t|	95% CI
Publication year			
G vs. A	2.38	0.140	−0.12–0.40
GG + AG vs. AA	1.86	0.205	−0.23–0.58
GG vs. AG + AA	1.03	0.411	−0.55–0.90
GG vs. AA	1.60	0.251	−0.40–0.88
MAF			
G vs. A	−3.03	0.094	−0.77–0.13
GG + AG vs. AA	−1.62	0.246	−1.14–0.51
GG vs. AG + AA	−3.09	0.091	−1.21–0.19
GG vs. AA	−2.70	0.114	−1.55–0.36

**Table 5:** Publication bias analysis of the meta-analysis.

	Test	t (95% CI)	P value
G vs. A	Begg’s Test		1.00
	Egger’s test	−0.24 (−237.14–212.55)	0.836
GG + AG vs. AA	Begg’s Test		0.308
	Egger’s test	5.39 (1.09–9.74)	0.033
GG vs. AG + AA	Begg’s Test		0.308
	Egger’s test	3.15 (−5.22–33.91)	0.087
GG vs. AA	Begg’s Test		0.734
	Egger’s test	−3.57 (−155.17–14.53)	0.070
