# Supplementary material for: Genetic association of RIT2 rs12456492 polymorphism and Parkinson’s disease susceptibility in Asian populations: a meta-analysis
Source: Sci Rep. 2015 Sep 3;5:13805. doi: 10.1038/srep13805 (PMC4558715; doi:10.1038/srep13805)
Supplement: Supplementary Figure S1 [file srep13805-s1.pdf]

Genetic association of RIT2 rs12456492 polymorphism and Parkinson's disease susceptibility in Asian populations: a meta-analysis

Yanjun Lu[1*], Wei Liu[2*], Kun Tan[3], Jing Peng[1], Yaowu Zhu[1], Xiong Wang[1#]

1. Department of Laboratory Medicine, Tongji Hospital, Tongji Medical College, Huazhong University of Science and Technology, Wuhan 430030, China.

2. Department of Public Health, Tongji Hospital, Tongji Medical College, Huazhong University of Science and Technology, Wuhan 430030, China.

3. Department of infection control, Tongji Hospital, Tongji Medical College, Huazhong University of Science and Technology, Wuhan 430030, China.

* Yanjun Lu and Wei Liu equally contributed to this work.

#Corresponding author: Xiong Wang, Email: tjhwangxiong@163.com

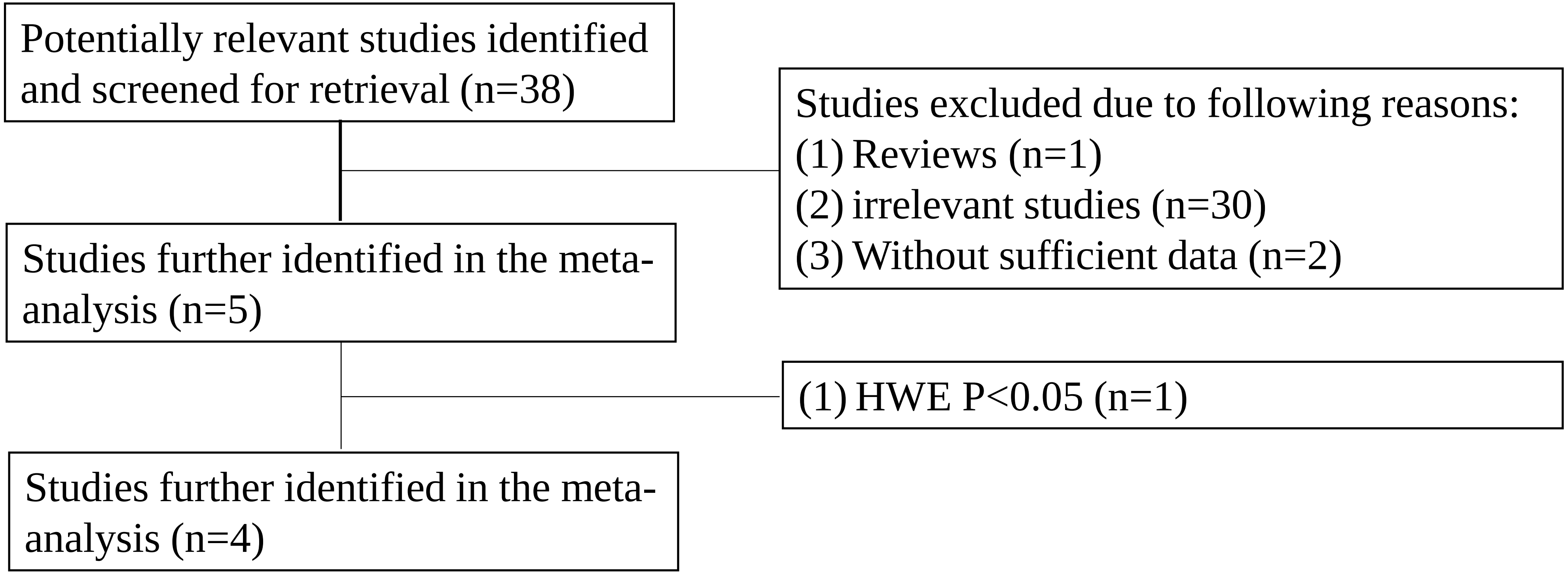

Flow diagram of literature search and selection.
